# Discharge transition experienced by older Korean women after hip fracture surgery: a qualitative study

**DOI:** 10.1186/s12912-021-00637-9

**Published:** 2021-06-28

**Authors:** Young Ji Ko, Ju Hee Lee, Seung-Hoon Baek

**Affiliations:** 1 Department of Nursing, Daegu Haany University, Daegu, South Korea; 2 College of Nursing, Yonsei University, Seoul, South Korea; 3 Department of Orthopedic Surgery, Kyungpook National University, Daegu, South Korea; 4 Department of Orthopedic Surgery, Kyungpook National University Hospital, Daegu, South Korea

**Keywords:** Hip fracture, Qualitative research, Nursing, Frail elderly, Patient discharge, Phase transition

## Abstract

**Background:**

This study aimed to explore older Korean women’s discharge transition experiences after hip fracture surgery.

**Methods:**

This was a descriptive qualitative study. Face-to-face interviews following hip fracture surgery were conducted on 12 women aged 65–87 years. Data were collected 1 to 2 days before discharge and again 4 weeks after discharge following hip fracture surgery, and were analyzed using qualitative content analysis.

**Results:**

Four main themes were identified: (1) challenge of discharge transition: unprepared discharge, transfer into other care settings, and eagerness for recovery; (2) physical and psychological distress against recovery: frail physical state and psychological difficulties; (3) dependent compliance: absolute trust in healthcare providers, indispensable support from the family, and passive participation in care; and (4) walking for things they took for granted: hope of walking and poor walking ability.

**Conclusions:**

After their hip fracture surgeries, older women hoped to be able to walk and perform simple daily chores they previously took for granted. Considering the physical and psychological frailty of older women undergoing hip surgery, systematic nursing interventions including collaboration and coordination with other healthcare professionals and settings are necessary to ensure the quality of continuous care during their post-surgery discharge transition. Encouraging partial weight bearing and initiating intervention to reduce fear of falling at the earliest possible time are essential to attain a stable discharge transition. Additionally, older women should be invited to participate in their care, and family involvement should be encouraged during the discharge transition period in South Korea.

## Introduction

With the rapid growth in the older population, hip fractures incidences are also increasing [1]. Fragility fractures of the hip are associated with higher morbidity, mortality, and socioeconomic burden [2], and those caused by simple falls with low impact occur four times more often in women than in men [3]. The standard treatment for such fractures is surgery [4]; however, short hospitalization periods after surgery increase concerns of older adults with complex health care needs [5]. Most older adults are transferred to other care settings after hip fracture surgery due to difficulties in walking or performing activities of daily living (ADL) [3, 6]. Transitions between care settings are burdensome [7], and older adults with various health problems are particularly vulnerable because they receive limited continuous care [8]. Even 1 year after surgery, many older adults fail to recover their preoperative performance levels in ADL [9]. Consequently, the concept of “discharge transition” is of particular significance in the care of older adults after hip fracture surgery.

## Background

The term “discharge” is defined as a transfer from one state or place to another [10]. A discharge transition period commonly begins prior to discharge and extends into the post-discharge period [11]. They have been identified as periods of heightened vulnerability for older adults [8]. In a previous study [5], 45.8% of the discharged older adults reported experiencing significant problems with managing their health care, including the performance of tasks associated with their health and adherence to ongoing care plans.

Hip fractures require particularly long periods to recover, and dependence is inevitable during a discharge transition [12]. The major transitional difficulties that older adults face after hip fractures are physical function limitations, and pain and its management [13]. Physical performance, including walking and ADL, is an essential prerequisite for older adults dwelling in a community environment [14]. Difficulties in physical performance in their life situations typically result in most older adults with various underlying diseases being transferred to other care settings [3, 13, 15, 16]. They perceive problems in the quality of care due to lack of a communication system between care settings [17], which causes limited continuous care [8]. Transitional care interventions, which include discharge planning and education, post-discharge follow-up, and care coordination, provided by healthcare providers for older patients have been widely implemented to ensure continuity of care. In a systematic evidence review [18], these interventions were shown to be effective in lowering mortality, emergency department visits, readmission rate and days.

Understanding older adults’ own views of their situation from the time of discharge transition is likely to be connected with their assessment of their care needs. Meleis’ transition theory [10], as a guiding framework, may provide a systematic understanding of their experiences during discharge transition. In particular, based on the transition theory [10], an in-depth understanding of transitional conditions facilitating or inhibiting the discharge transition would affect the quality of nursing therapeutics for a healthy transition. The transitional conditions include individual conditions, such as readiness and knowledge, and environmental conditions, such as family or healthcare providers’ support [10]. Qualitative approaches are appropriate to fully understand the experience of discharge transition from the perspective of older adults [19, 20]. Some previous qualitative studies related to older adults with hip fracture surgery have described the experience of early discharge [21], pain and rehabilitation after discharge [16], and recovery after hip fracture surgery [12, 20, 22]. To the best of our knowledge, only a few studies have described older adults’ discharge transition experiences in Korea.

There are also differences between Eastern and Western health care systems for care after hip fracture surgery. In South Korea, surgical hospitals provide postoperative care and rehabilitation during short hospitalization [6], while in the United States, patients are systematically transferred to post-acute care facilities for rehabilitation after discharge from surgical hospitals [23]. In addition to the differences in the health care systems, the recovery experiences of older adults after hip fracture surgery in Asia will differ from those in the Western countries, given that in the family-oriented Asian cultures, the family has the most influence on the patient’s life [24, 25]. In a study conducted in Taiwan [26], it was reported that emotional support predicted physical function and health-related quality of life of older adults living with extended family members after hip fracture surgery. Strong bonds among family members may enhance the significance of emotional support for recovery.

Owing to the rapid growth of the older population, the annual incidence of hip fractures in Korea is approximately 82,550 cases, and older adults accounted for 64,366 (78%) of these cases [27]. Therefore, there is an urgent need to explore the experiences of older adults after hip fracture surgery. The purpose of this study was to describe older adults’ experiences and perceptions of their transitional conditions during their discharge transitions after hip fracture surgery in South Korea.

## Method

### Design

This was a descriptive qualitative study based on semi-structured interviews using open-ended questions.

### Participants

The participants comprised 12 older women aged 65–87 years (median age 77.5 years) who did not have any serious neurological disease, such as stroke or dementia with paralysis and/or cognitive deficits. Only those older adults who understood the study purpose and agreed to participate were recruited in the study. The participants were recruited using convenience sampling from a teaching university hospital in South Korea (Table 1). The number of comorbidities ranged from 2 to 6, with a median of 2.5, except a participant with no comorbidity. All participants were female. Seven participants walked indoors and outdoors independently, while five participants walked indoors and outdoors using walking aids before hip fracture. Most participants (10 people) were transferred into other care settings, including long-care hospitals and rehabilitation hospitals. The participants’ primary caregivers were all family members.

**Table 1:** Demographics of older patients and primary caregivers

	Age (years)	Walking status before hip surgery	No. of comorbidities	Type of hip surgery	Residence after discharge	Primary caregiver	Interviewed after discharge	Walking status after discharge
001	78	Independent walking in and outdoors	2	Internal fixation	Rehabilitation hospital	Daughter	Yes	Immobile status in and outdoors
002	65	Independent walking in and outdoors	0	Total hip replacement	Home	Younger Sister	Yes	Independent walking indoors; using a walking aid outdoors
003	87	Independent walking indoors; using a walking aid outdoors	5	Internal fixation	Long term care hospital	Daughter in law	No	Immobile status in and outdoors
004	75	Independent walking indoors; using a walking aid outdoors	6	Internal fixation	Long term care hospital	Husband	Yes	Immobile status in and outdoors
005	80	Independent walking in and outdoors	4	Internal fixation	Rehabilitation hospital	Son	Yes	Using a walking aid indoors
006	65	Independent walking indoors; using a walking aid outdoors	3	Total hip replacement	Rehabilitation hospital	Husband	Yes	Immobile status in and outdoors
007	73	Independent walking in and outdoors	2	Hemiarthroplasty	Rehabilitation hospital	Daughter	No	Immobile status in and outdoors
008	85	Independent walking in and outdoors	5	Internal fixation	Long term care hospital	Son	Yes	Immobile status in and outdoors
009	78	Independent walking in and outdoors	2	Total hip replacement	Long term care hospital	Son	Yes	Immobile status in and outdoors
010	74	Independent walking in and outdoors	2	Total hip replacement	Long term care hospital	Husband	Yes	Using a walking aid outdoors
011	81	Using a walking aid in and outdoors	4	Internal fixation	Long term care hospital	Granddaughter	No	Death
012	77	Using a walking aid in and outdoors	2	Internal fixation	Home	Daughter	Yes	Using a walking aid in and outdoors

### Data collection

From November 2017 to February 2018, data were collected 1 to 2 days before discharge, and again 4 weeks after discharge, considering the time span of the discharge transition that begins prior to discharge and extends into the post-discharge period [11]. Of the 12 participants, 3 could not participate in the follow-up interview because of death (one participant) and loss of follow-up (two participants). Thus, 12 participants were included in the pre-discharge survey and 9 in the post-discharge survey. Pre-discharge interviews were conducted face-to-face in hospital wards. Four weeks after discharge, each older woman was individually interviewed in an outpatient meeting room. Data collection was carefully conducted using an interview guide with open-ended and semi-structured questions. The main question was about the older women’s perception of transitional conditions facilitating or inhibiting healthy transitions as a dimension of Meleis’ transition theory [10] during discharge transition. Each interview began with the general question, “Tell me about your discharge experience.” We also inquired about the perceived difficulties during discharge transitions, factors that promote or impede recovery, and participants’ needs (Table 2). The interview guide was suggested as a supplementary file.

**Table 2:** Interview topic guide

< 1 or 2 days before discharge>	
1) Describe your experience including readiness during discharge periods.	
2) What are barriers you have perceived during discharge periods?	
3) What are facilitators you have perceived during discharge periods?	
4) What efforts do you make for healthy discharge transition?	
5) Tell me information, education, support to want to provide.	
6) Is there anything else you feel is important to mention?	
< average 4 weeks after discharge>	
1) Describe your experience including activities of daily living after discharge.	
2) What are barriers you have perceived after discharge periods?	
3) What are facilitators you have perceived after discharge periods?	
4) Describe any change or difference after discharge periods.	
5) What efforts do you make for adaptation of activities of daily living?	
6) Tell me information, education, support to want to provide.	
7) Is there anything else you feel is important to mention?	

All the interviews were conducted by the first author, who has attended qualitative research classes and related workshops and has experience in conducting qualitative research. A research assistant played an auxiliary role including recording the interviews. The time required for each individual interview was approximately 30–40 min, and all interviews were audio-recorded with the participants’ consent. The recorded interviews were only accessible to the interviewers, and the participants were informed that the recordings would be deleted immediately after the research. Twelve participants were interviewed to ensure that data saturation was reached; that is, no new information was obtained from a diverse range of statements. The researchers carefully listened to the participants and recorded meaningful statements as written notes during the interviews. Additionally, the researchers asked further questions to clarify whether the participants’ statements were ambiguous. Recorded material was transcribed verbatim by a research assistant trained in qualitative data analysis. Transcribed interview data and field notes were then analyzed.

### Data analysis

The qualitative data were analyzed through content analysis [28] using NVivo 11 (QSR International, Melbourne, Australia). Meaningful data were extracted after reading the transcribed interviews and field notes several times and were coded through constant comparative analysis [29] to identify similarities and differences between data segments. This work was mainly conducted by the first author in close cooperation and discussion with the co-authors of this study. Through several discussions, the coded data were classified based on similarities and integrated into categories. The commonalities and natural changes in the data were identified and themes were derived as abstract entities from the categories. We repeated the process of reading and reviewing the original data again to clarify the relevance of the derived categories and key themes, which were refined, as necessary.

### Ethical considerations

This study complied with the principles laid down in the Declaration of Helsinki and was approved by the institutional review board (Daegu Korean Medicine Hospital of Daegu Haany University, DHUMC-D-17024-PRO-02). Informed consent was provided and obtained from all participants before study commencement. Confidentiality and privacy were guaranteed and each interview file was coded and anonymized.

### Rigor

The trustworthiness of this study was established through prolonged engagement, triangulation of data sources, peer review, detailed description, and external auditing [30]. To assure credibility, participants were interviewed twice, and an additional telephone interview was conducted with two participants to make sense of the data. This allowed relationships to be built between the interviewer and the interviewees and provided an opportunity to revise any misinformation.

Concerning data triangulation, we compared the interview transcripts with the field notes. For peer review, the second author, who is experienced in qualitative research, repeatedly reviewed the results to ensure the quality and validity of the analysis at all stages. All authors discussed the findings until an agreement was reached. To enhance transferability, we provided a detailed description of the setting, participants’ characteristics, and the selection methods. To ensure dependability and confirmability, a professor of nursing with substantial experience in qualitative research examined the process, analyses, and findings. The Consolidated Criteria for Reporting Qualitative Research (COREQ) checklist was used to report the findings [31].

## Results

The analysis yielded four main themes and ten categories. Each theme is illustrated using excerpts from the interviews.

### Theme 1: challenge of discharge transition

#### Unprepared discharge

Participants recognized their unstable state at the time of discharge and wanted to stay longer in the surgery hospital. Older adults (5 of 12) were forced to complete discharge from the surgery hospital despite their limited mobility. They believed that the recoveries were delayed owing to the short hospitalization period after surgery. They expressed feeling upset at being discharged so soon.

*I was discharged after a mere four and a half days; so, I felt [I received] unfair treatment. How [can you discharge] a person who has [had] a major operation so soon! That’s sad* (Interviewee 8).

#### Transfer into other care settings

Most older adults (10 of 12) moved to another care setting after discharge. Four participants faced difficulties in finding their own care setting without help and lacked pertinent information. Participants who moved to other care settings reported that they received care unrelated to the therapeutic instructions.

*I said I could exercise, but they said, “no.” When I asked why not, they told me that they would not allow me to do it because they were in a situation where they had to take responsibility about (anything) going wrong* (Interviewee 6).

Some participants (four older adults) had to alter their living environment because of mobility limitations.

*My house is on the fourth floor; so, the thing to worry about now is whether I have to move to a house with an elevator* (Interviewee 9).

#### Eagerness for recovery

Older adults (6 of 12) showed eagerness for recovery to cope with the challenges of their discharge transitions. They commented on nutritional management, rehabilitative exercise, walking, and adhering to physician’s orders*.*

*I have to walk and turn around using a walker now. I have to focus on this. I’m ready to make recovery quickly. I have to exercise, eat well, and get better every day* (Interviewee 12).

Two participants tried to understand and accept their diseases instead of denying them to improve their recovery process.

*When I think that even young people fall and come to the hospital, I feel that the same can happen to me! Anyone can experience this! It is nice to have a new artificial joint inserted. After the surgery, I encouraged myself to get along with the artificial joint* (Interviewee 2).

### Theme 2: physical and psychological distress against recovery

#### Frail physical state

Participants were in a frail physical state. Ten out of the twelve participants had multiple or severe underlying diseases including osteoporosis. They were using polypharmacy before the fracture owing to serious underlying diseases and had poor health status. Severe underlying diseases affected the patients’ recovery processes after hip fracture surgery, and they continued to be in a poor state after discharge.

*I have diabetes, poor kidneys, higher blood pressure, and bad eyes. My diabetes has been around for 50 years. Only 30% of my kidneys remain* (Interviewee 8)*.*

They did not eat enough owing to a limited appetite, digestion, or diet due to their underlying diseases.

*Actually, I do not eat well. I say, “I do not want to eat” when my child asks me what I want to eat. Whenever I try to eat, I get tired easily. I’m going to ask for some medicine [to improve my] appetite* (Interviewee 5).

#### Psychological difficulties

Some participants also experienced psychological difficulties. Their frail physical states negatively affected their confidence in recovery. The worse their physical frailness, the more they fell into despair (6 of 12), which hindered their recovery. The following interviewee was informed that her walking should be limited for a month due to severe osteoporosis, and she showed frustration related to movement restriction.

*I [was] unpleasantly surprised when I heard that I should not walk for a month or so. I knew I would walk if it is possible to walk little by little. Then, I wanted to go to the bathroom. I wanted to do it gradually. I took a leak and shat in my bed without moving. Oh! How miserable my life has become!* (Interviewee 4).

Other older adults (2 of 12) perceived their current health conditions as a punishment.

*“Did I commit [a] sin?” People said repeatedly “it is not a sin.” … Getting sick like this? I think I am being punished* (Interviewee 9).

### Theme 3: dependent compliance

#### Absolute trust in healthcare providers

Participants sought care at a tertiary hospital either because the secondary hospitals were reluctant to perform surgery on patients who may have underlying diseases, or because they had successful experiences—directly or indirectly—at tertiary hospitals. Most participants (10 of 12) expressed gratitude for undergoing surgery and were satisfied with their surgeries.

*What if I get broken and do not undergo surgery? I’m feeling good because I underwent surgery* (Interviewee 12).

Older adults (6 of 12) showed strong trust in healthcare providers and noted their willingness to follow their therapeutic instructions.

*All I can do is listen to the doctor’s words and do what they say. There is only that* (Interviewee 10).

#### Indispensable support from the family

All participants received support from family members throughout their discharge transitions. Some (2 of 12) had home-visit care services including support in physical and household activities in their homes. This help was also available to older adults. However, they could not continue their daily lives without family support. Family members were primary caregivers who mainly helped with activities of daily life and encouraged exercise and diet.

*My son brought some meat for me, saying I should eat beef, beef, beef. I should eat because of my son. I feel like [shedding] a tear when I think of my son* (Interviewee 5).

Older adults felt grateful for family support, while some older adults (3 of 12) also felt the burden of being cared for.

*I feel sorry for my child. My child would not be suffering this way had I died early. I’m worried about how much the hospital bill will be* (Interviewee 8).

#### Passive participation in care

Most participants (8 of 12) received insufficient information about postoperative management owing to staff members’ busy schedules or lack of interest. Some participants had difficulty in reading text-based discharge information.

*Even if my son asked me to keep reading, I could not read the text because it was difficult to read* (Interviewee 9).

Nevertheless, they expressed a vague expectation that it would get better with time. Some medical staff explained the surgery process to the caregivers instead of the older adults.

*I do not know much about it because my daughter is the one who understands* (Interviewee 7).

### Theme 4: walking for things they took for granted

#### Hope of walking

Half of the participants (6 of 12) experienced physical recovery, while others did not. Physical recovery indicated reduced pain, improved physical condition, and starting to move little by little.

*I have a little more energy now than when I was discharged, and the hips hurt less*. (Interviewee 11).

Most participants (9 of 12) described that being able to walk was their main wish during the discharge transition. Their expectations for walking ranged from using the toilet independently or with walking aids, returning to their homes, to going to the farm according to their physical recovery statuses.

*Can I walk or not, getting hurt like this? Walking around on my feet. Right, it would be better if I go to the bathroom with my feet* (Interviewee 4).

The purpose of improving the walking ability was that participants could perform simple daily chores. Performing tasks they had previously taken for granted was an essential part of daily life.

*I just want to go home and cook for the poor youngest* (Interviewee 1).

#### Poor walking ability

Despite their hopes of walking, their walking ability was poor during the discharge transition period; some older adults (4 of 12) were mobile indoors or outdoors only when using walking aids, while most (7 of 12) were immobile both indoors and outdoors. In addition to their frail physical state, there were some direct factors affecting walking ability. Severe pain hindered their recovery (7 of 12). Most older adults (9 of 12) were also afraid to move owing to fear of falling and subsequent re-fracture, even though their pain had significantly receded.

*There is nothing painful now, but I’m afraid to take a step. I can’t take a step. I do not think I can get up* (Interviewee 9).

## Discussion

The purpose of this study was to clarify older women’s discharge transition experiences after hip fracture surgery. We identified four main themes—challenge of discharge transition, physical and psychological distress against recovery, dependent compliance, and walking for things they took for granted. This study is significant as it elucidates patients’ distinct experiences, which inform future discharge transition programs.

First, the challenges of their discharge transition included three categories—unprepared discharge, transfer into other care settings, and eagerness for recovery. Consistent with a prior study [15], most older women believed that their state was unstable and wanted to stay longer in the surgery hospital. Short hospitalization as a measure for cost-saving is currently the rule rather than the exception. However, short hospitalization within 10 days for patients with hip fracture is associated with higher one-year mortality after discharge in South Korea [6]. In addition, postoperative care and rehabilitation at surgical hospitals during short hospitalization is unlikely to be sufficient to recover. In this study, most participants were transferred to long-care and rehabilitation hospitals after discharge; this finding was compared with a study [6] that most older adults were transferred to home or other non-medical settings. However, older patients transferred to long-care hospitals could not receive rehabilitation because long-care facilities focus more on patient safety rather than care to promote physical function [32]. Limited continuous care makes it difficult for older women’s needs to be met [11] and restricted rehabilitative care causes higher morbidity and mortality [33].

To facilitate safe discharge transition, communication and collaboration with healthcare professionals and ensuring adequate care settings are important roles in nursing [17, 34]. In addition, in a previous study [35], nurse-led rehabilitative practices were reported to reduce functional decline for older adults that have undergone hip fracture surgery. Thus, it is necessary for nurses to not only to recognize the importance of rehabilitative care but also establish rehabilitative strategies. The current finding that some older women struggled to recover functions such as exercising and eating was consistent with a previous study [12], which was indicated that older adults began to plan for their recoveries immediately after hip fracture surgeries because regaining independence was the most important issue in their daily lives [12].

As a second theme, physical and psychological distress against recovery was indicated. Older women with underlying diseases were in a frail physical state and were malnourished owing to poor appetite, digestion, and limited diet due to their underlying diseases, even though they recognized the importance of food intake [36]. The frailty likely made their recovery more difficult. Hip fracture in older adults reflects the loss of skeletal strength from osteoporosis [3], and rate of occurrence is higher for older women than for older men [37]. Osteoporosis, which most older adults with hip fracture have, is closely correlated with sarcopenia. The combination of osteoporosis and sarcopenia may cause hip fracture [38, 39]. Thus, interventions for sarcopenia as well as osteoporosis are essential for improving the frail physical states of older adults, including exercise, nutritional support, and pharmacological treatments [38, 39].

Further, frail physical state caused psychological distress including loss of confidence and frustration. This was consistent with a previous study [40], which demonstrated that frailty was the most important prognostic factor for depression and anxiety in patients with hip fracture. Psychological distress plays a major role in interfering with treatment after hip fracture [40, 41]. To overcome discharge transition instability, not only physical, but also psychological support may prove helpful. According to a previous study [42], older women living alone in Japan showed lower risk of psychological distress when they had relationships with other people, including family. Relationships with family for older women after hip fracture surgery are connected with the third theme.

The third theme, dependent compliance, refers to absolute trust in healthcare providers, indispensable support from the family, and passive participation in care. The result regarding absolute trust in healthcare providers was similar to that of a previous study [12] in which older adults expressed their need to feel supported by healthcare providers. Older women also depended entirely on family members, likely because of the Korean family-centered culture [43] and their limited social interaction [21], although some had home-visit care services during limited hours in their home.

Older women were rather passive participants in their own care. They were either partly or not at all involved in the planning of their treatment and recovery because managing their own illnesses was complex and challenging [8]. In Eastern cultures, physicians often tend to explain care plans to the family rather than individual patients [43]. In this study, older women wanted to feel supported and at the same time found it difficult to participate in their own care. Feeling supported is likely to be achieved when nurses invite older women to participate in their care [8, 43]. Family involvement is also important in family-oriented cultures [42, 43]. Thus, inviting the patients (older women in this case) and their families to participate in the formers’ postoperative care should be considered at the discharge transition stage in South Korea.

The final theme was walking for things they took for granted. The purpose of walking during recovery was to perform simple daily chores. This is consistent with a previous study demonstrating that an essential requirement for daily functioning is recovery walking [44]. Their expectations for walking differed according to their ambulation states. According to past studies, the walking abilities of older women after their hip fracture surgeries was affected by various factors: underlying diseases [45, 46], pre-fracture mobility [45, 46], age [46], and health status [47], which is similar with the finding of this study.

In addition to the physical and psychological distress mentioned above, in this study, severe pain and fear of falling were found to act as direct factors affecting walking ability. Pain is well recognized to be a major barrier for early recovery and should thus be treated as soon as possible [48]. However, following hip fracture surgery, older adults are often not actively treated for pain during their hospitalization because of the difficulty of assessment and concerns regarding drug complications [49]. Tailored pain management is needed for safe and adequate pain relief [48, 49], considering that prolonged pain was independently associated with a catastrophic decline in walking recovery [44].

Older women also complained of fear of falling at the onset of the recovery period. A previous study [50] reported that the fear of falling was the primary constraint in the poorest ADL group and instrumental ADL group. Older adults continued to complain of fear of falling even 3–6 months after hip fracture surgery [50], and after completion of a 4–6 weeks rehabilitation program [51]. Fear of falling was also more frequent in women than in men [52]. Considering that fear of falling is a factor that hinders walking ability from the onset, assessments and interventions to reduce fear of falling should be initiated as soon as possible after surgery.

In this study, most participants were immobile both indoors and outdoors during the discharge transition periods even though they were mobile before their fractures. According to a study [3], most older adults have difficulty walking at discharge after hip fracture surgery and their functioning does not return to the pre-fracture state even 1 year after surgery [9]. Delayed partial weight bearing after surgery has been reported to predict walking failure after hip fracture surgery [45]. For effective physical rehabilitation including partial weight bearing at the earliest possible time, nursing needs to collaborate and coordinate with interdisciplinary personnel [17, 34].

### Limitations

This study had several limitations. First, the sample was recruited from a tertiary teaching hospital in Korea using convenience sampling; thus, our results may not be generalizable. Further study can investigate other differences in older women treated in non-tertiary hospitals. Second, all older adults in our study were women. Although hip fractures caused by simple falls are four times more common in women than in men [3], it is necessary to examine whether gender differences exist in their discharge transition experiences after hip fracture surgery. Third, our sample size was small. Moreover, three of the selected participants could not participate after discharge, as one of them passed away and the other two could not be reached. This variance may have affected the results. Lastly, our findings cannot be extended to countries with different healthcare systems and cultures.

## Conclusion

Knowledge about older women’s discharge transition experiences after hip fracture surgery could help nurses improve transitional care in acute settings. After their hip surgeries, older women hoped to be able to walk so as to perform the simple daily chores that they previously took for granted. Considering the physical and psychological frailty of older women undergoing hip surgery, systematic nursing interventions including collaboration and coordination with other healthcare professionals and settings are necessary to ensure the quality of continuous care during discharge transition periods after hip fracture surgery. Encouraging partial weight bearing and initiating intervention to reduce fear of falling at the earliest possible time are essential to attaining a stable discharge transition. Additionally, the older women should be invited to participate in their care and family involvement should be encouraged during the discharge transition period in South Korea. We propose a follow-up study to evaluate older adults’ discharge transition experiences by increasing post-discharge periods, considering the inevitability of long-term recovery after hip fracture surgery.

## Data Availability

Data is available upon reasonable request from the first and corresponding authors.

## Abbreviation

ADL: Activities of daily living
